# Exploiting the PIR Sensor Analog Behavior as Thermoreceptor: Movement Direction Classification Based on Spiking Neurons

**DOI:** 10.3390/s23135816

**Published:** 2023-06-22

**Authors:** Jose-Maria Guerrero-Rodriguez, Maria-Angeles Cifredo-Chacon, Clemente Cobos Sánchez, Fernando Perez-Peña

**Affiliations:** 1 Microelectronic Circuit Design Group, Engineering School, University of Cadiz, Campus Universitario de Puerto Real, Avda. Universidad de Cádiz, nº 10, CP 11519 Puerto Real, Cádiz, Spain; josem.guerrero@uca.es (J.-M.G.-R.); clemente.cobos@uca.es (C.C.S.); 2 Applied Robotics Lab, Engineering School, University of Cadiz, Campus Universitario de Puerto Real, Avda. Universidad de Cádiz, nº 10, CP 11519 Puerto Real, Cádiz, Spain; fernandoperez.pena@uca.es

**Keywords:** PIR passive infrared sensor, spiking neural network, optical flow, human occupancy detection, people detection

## Abstract

Pyroelectric infrared sensors (PIR) are widely used as infrared (IR) detectors due to their basic implementation, low cost, low power, and performance. Combined with a Fresnel lens, they can be used as a binary detector in applications of presence and motion control. Furthermore, due to their features, they can be used in autonomous intelligent devices or included in robotics applications or sensor networks. In this work, two neural processing architectures are presented: (1) an analog processing approach to achieve the behavior of a presynaptic neuron from a PIR sensor. An analog circuit similar to the leaky integrate and fire model is implemented to be able to generate spiking rates proportional to the IR stimuli received at a PIR sensor. (2) An embedded postsynaptic neuron where a spiking neural network matrix together with an algorithm based on digital processing techniques is introduced. This structure allows connecting a set of sensors to the post-synaptic circuit emulating an optic nerve. As a case study, the entire neural processing approach presented in this paper is applied to optical flow detection considering a four-PIR array as input. The results validate both the spiking approach for an analog sensor presented and the ability to retrieve the analog information sent as spike trains in a simulated optic nerve.

## 1. Introduction

Whether in the interest of personal safety, access control, management, or energy saving, occupancy behavior (OB) and energy management techniques are gaining momentum in today’s intelligent detection applications for living spaces and buildings.

Currently, GPS/POI data [1] has been widely used for outdoor localization; for indoor localization wireless technologies (BLE [2], WIFI [3], and RFID [4]), as well as hybrid approaches like sensor fusion [5] techniques, have been popular approaches for the purposes of occupancy localization, tracking, and monitoring. However, particularly considering cases for indoor OB analysis, the thermal sensor for people detection and tracking is widely used. Detection of people, human occupancy, or intrusion to a restricted area is facilitated since the human body actuates as an IR radiator. This phenomenon can be analyzed as the heat emission by the human body in the form of black-body radiation (3–50 μm with a max peak of about 9.4 μm).

Several methods are available to detect heat by using the emission of electromagnetic radiation from hot surfaces and Stefan–Boltzmann law, being thermopiles, bolometers, and pyrometer detectors the most used devices [6]. The last cited is technically known and presented as PIR (Passive Infrared, or in other contexts, pyroelectric infrared) motion detectors. It is based on the pyroelectricity phenomena and is commercially available as a single element, accompanied by a Fresnel lens.

These devices have gained popularity due to the massive development of low-cost integrated systems dedicated to localization and occupancy detection of humans or animals, moving within their field of view (FoV) and a delimited area.

For this, different authors have reported the use of PIR sensors as candidates to monitor the presence of people utilizing microcontrollers or personal computers as final processing devices and to display the results [7,8]. Yang et al. [9] examine general methods to ensure the correct people detection considering the PIR output voltage to different cases, analyzing the generated waveforms of the human body thermal infrared signal, and also examining the techniques for people identification. Thermal sensors have also been used for gesture recognition applications [10]. Wojtczuk et al. [11] present a 4 × 4 PIR sensor array to obtain a pattern from infrared sensors leading to the classification of some hand patterns.

In general, these articles consider the use of the thermo sensor together with its Fresnel lens, either employing its output analogical or utilizing the discretized one and then, consequently, applying a window comparator circuit to analog measurement to finally give a discrete response.

A minor number of researchers have focused their works on thermal sensor arrays. Cameras and image sensors for viewing thermal images can currently be found on the market at reasonable prices.

In addition, the use of enhanced resolution IR arrays is contrary to the people’s privacy preservation present in the space under study. This is especially important in the people’s detection of indoor non-public or personal use areas [12]. In this sense, diverse commercial integrated IR sensor arrays allow us to capture a low-res thermal image, such as the Panasonic^®^ AMG88xx Series (Grid-Eye^®^ Infrared Array Sensors) or Omron^®^ D6T Series MEMS Thermal Sensors.

In the analysis of Sirmacek et al. [13], the work aims to explore low-cost and privacy-preserving solutions for occupancy prediction utilizing an 8 × 8 commercial sensor to explore the room from the ceiling. Narayana et al. [14] compare his proposed PIR array (a two-element set including the Fresnel lens) with a Grid-Eye sensor and a Melexis thermopile detector. The PIR sensor arrangement reaches a greater detection distance (up to 20 m) regarding the Panasonic thermopile sensor, around 5 m. They apply an analog procedure to the output of the PIR sensor response but maintain the utilization of the Fresnel lens. Even with the disadvantage of the low resolution of the PIR sensor and Fresnel lens, these devices have a better position regarding cost, range, power consumption, and response rate concerning integrated thermopile alternatives (with some exceptions [15]).

Taking advantage of the properties of PIR sensors, other works have proposed PIRs element arrays assembled from a two-dimensional arrangement of simple detector devices including or not including the corresponding Fresnel lens [16,17,18,19,20]. Petrova and Spasov’s work provides a comparison of different sensing procedures using low-resolution IR sensors for indoor detection, localization, and people counting concerning the IR sensor resolution and the processing methods and techniques [21].

Different authors have suggested a discrete PIR array as an alternative to people detection, as in [22], and localized arrays as in [23,24,25]. Wu et al. [26] present an interesting image sensor based on a PIR and Fresnel lens. A chopper mechanism on top of the sensor is included to act as a basic optical scan of the scene.

Various reports also suggest the advantage of machine learning methods to obtain occupancy and counting conclusions from PIR-type sensors [16,27,28,29,30]. On the other hand, ref. [31] proposed the use of the transferable believe model (TBM) along with the Kalman filter to estimate the behavior of several sets of arrays of four PIR sensors (with Fresnel lenses) following the subjects from the ceiling. Works on the application of data fusion techniques to determine the identification of persons are also known (previously cited [25]).

### A Bioinspired Focus of the PIR Sensors Application

Bio-inspired engineering, in general, is the application of biomimicry (the use of natural models, systems, and elements for human innovations) to aid design, performance improvement, or research of the operating principles of biological systems. In the case of sensors and detectors, it is a known trend due to its direct application to measurement systems and robotics.

**Figure 1:**
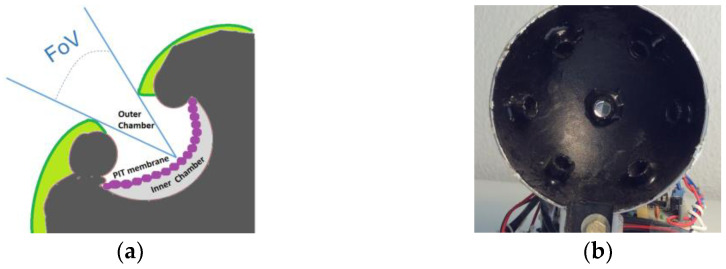
(**a**) PIT organ of the snake: structure similar to the pre-evolved eye, pin-hole lens based. (**b**) A PIT-inspired image sensor, implemented with a seven PIRs array (from authors).

Regarding infrared (IR) detection, Campbell [32] has analyzed the existence of thermoreceptors in different animals or insects. Mainly, the IR vision is supported by the Pit Organ (Figure 1a) or antennas (in the insect cases). This perception of the temperature in animals is known as thermoception or thermoreception. This current of inspiration has also revolutionized research into materials and technologies to detect IR radiation using nature’s methods [33,34]. Nevertheless, Clark et al. [35] report that biological thermosense organs are not able to resolve some detailed spatial patterns, especially the detection of objects with significant temperature contrast moving across homogeneous thermal backgrounds. But the present application does not require that level of resolution.

However, PIR sensor usage offers advantages due to their low-cost, simple design, and fast electric response for hot sources location purposes. The electrical response of a PIR-type sensor is analog, presenting a positive impulse according to the temperature detected (or when a hot object approaches the detector). In this case, the sensor suffers a thermal energy increment to respect the background temperature. In the same way, a negative impulse corresponds to the decrease in temperature in the sensor device regarding the environment temperature or when any hot body goes out of the FoV. This analog behavior allows us a comparative study from the point of view of spiking neurons to the PIR sensor as a sensory cell.

The approach of current neural networks is carried out by applying the behavior of biological neurons to networks of artificial neurons. The interconnection of spiking neurons lets us implement complex artificial neural networks. This paradigm is considered the third generation of neural networks, preceded by McCulloch–Pitts neuron discrete (or ‘first generation’ of artificial neurons based uniquely on the threshold as activation function) and the artificial neural networks (ANN) with continuous activations, like sigmoidal or hyperbolic tangents functions (‘second generation’), that are commonly implemented using hardware or software technologies.

About the theoretical and practical aspects of neural networks, particularly spiking neurons, exist an excessive number of bibliographical references documented by a notable group of authors. A complete introduction to spiking networks can be consulted in the Maass-Bishop edited book [36].

The aims of this work are the analysis and design of the necessary hardware to carry out processing with spiking techniques that allow the implementation of elementary applications to detect, monitor, and track people within a defined area. For this purpose, a spatial matrix arrangement of PIR sensors and classical digital processing routines are used. This approach allows the signals from the individual sensor elements to be carried by a single wire to the central processor. The argument is that the analog reaction of the PIR sensor, at its base, presents a behavior comparable to the biological conduct of sensitive cells due to its impulsive electrical response to the charge/discharge of a capacitor. Only to fix objectives, this brief study is made on the concept of bioinspired and the infrared vision of some animals.

However, our aim is focused on low-resolution image sensors with the interest of using them in applications that do not allow the identification of subjects or to be carried on micro-robots where energy is limited. We are also facing weight restrictions and low rates of local data image processing due to small microcontroller capabilities.

**Figure 2:**
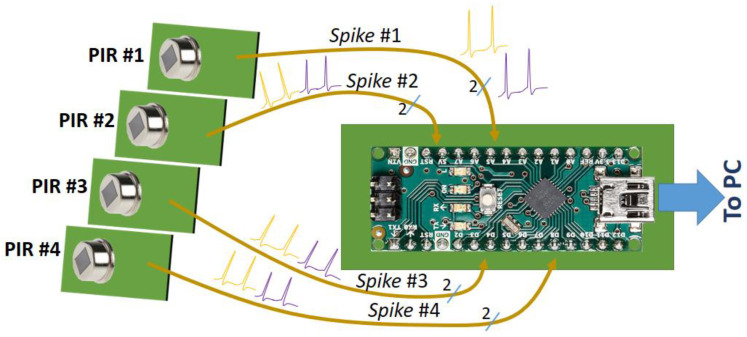
Project purpose: Design and implementation of PIR sensor arrays with the intent of people tracking or occupancy counter. Each sensor module transmits through a pair of cables and two different spiking sequences toward the main processor board: when the hot body approaches the sensor and as the body moves away.

A specific electronic design of the PIR assembly to obtain analog signals for a microcontroller-embedded processor (Figure 2) was considered, and the IRA-E700 PIR sensors [37] from Murata© (Kyoto, Japan) were chosen. They exhibit high sensitivity and reliable performance. IRA-E700 series also present a reasonable ratio of price and benefits.

Since the problem is approached from an analog perspective, to continue the processing chain the typical inclusion of Fresnel lenses has been avoided. Now the sensor requires the use of a single pyro-element to work.

Our highlighted contributions are mainly:

- Analog use of sensor without Fresnel lens;
- The design and development of a circuit to process the PIR detector as a sensitive spiking cell;
- A procedure based on digital signal processing techniques, using digital filter implementation to obtain fast algorithms;
- An arrangement of array-type sensors to convert them into very low-resolution image sensors, but with the ability to process optical flow.

This article reviews several research projects, the state of the art of PIR sensors as a single device, and applications of PIR array sensors (Section 1). The rest of the paper is outlined as follows: a description of the sensor PIR physical concepts as the hardware methods and software procedures utilized to carry out the sensor IR proposal (Section 2). In Section 3, the article presents a study case: a basic optical flow implementation based on the hardware and processing techniques proposed. Next, Section 4 (Results) describes any measurements of human activity detection, flow detection, and performances of the suggested optical flow application. Finally, Section 5 (Discussion) presents the advantages and objections to this contribution, and concludes in Section 6.

## 2. Materials and Methods

### 2.1. The PIR Sensors and Spiking Sensory Cell Emulation

A pyroelectric infrared sensor is a category of thermal detectors that responds to the change in the sensor’s temperature from an emitted electromagnetic energy and the law of radiation according to Stefan and Boltzmann law. The physical principles of operation of pyroelectric components and the different PIR detector types have been profusely discussed in the scientific literature [14,38,39,40]. It is also possible to find an approximation of its operation in the manufacturer’s catalogs of these devices [41,42].

The Al-Jazzar et al. paper [43] shows a good relationship between sensor physics, heat flow, and the geometry of the optical field of view. Thang et al. [44] have presented a complete study to develop and construct an experimental setup for defining the optic problem when PIR sensors are utilized (sensibility, resolution, position, and centering error).

**Figure 3:**
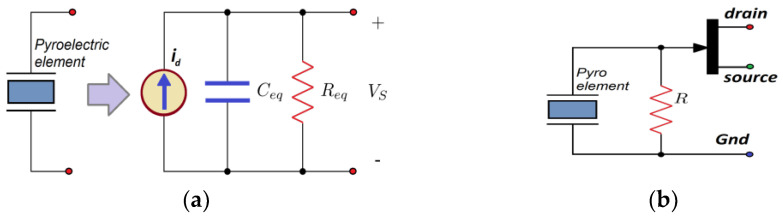
A single-element PIR sensor: (**a**) the pyro-crystal modeled. (**b**) Typical commercial PIR device including a FET amplifier integrated into the encapsulate.

The fundamental PIR detector type is based on a single-element sensing crystal. The pyroelectric device converts the incident thermal radiation into an electrical signal across the conductive plates. This sensor may be modeled as a capacitor, where the charge concentration is a function of the temperature (Figure 3a). For this reason, a dependent current source is included to simulate the changes in the material. Then, *i_p_* represents the current flowing through the device on which an incident thermal radiation causes a temperature change. The equation to define the output voltage is:

(1) $$V_{s}=R_{eq}i_{p}=R_{eq}pA_{s}\frac{dT_{p}}{dt}$$

where *p* is the pyroelectric constant and depends on the material, and *A_s_* is the area of the sensitive elements of the detector. The capacitance created by the electric field detector plates is modeled as a capacitor *C_eq_* with the pyroelectric crystal as dielectric. *R_eq_* is the equivalent resistance from pyroelectric crystal and input impedance necessary to fix in the pre-amplifier.

The measuring principle is based on the polarization changes of the pyroelectric material, actuating as a capacitor. Therefore, significant changes in the net charge are generated depending on the increments and decrements of the crystal’s temperature. In consequence, in the case of permanent stimulation, the sensor does not present a permanent output of current or voltage. This analog behavior allows a close comparison to the behavior of the biological neuron.

On the other hand, the thermal cell is also very dependent on internal temperature changes. For this reason, it is usually to encapsulate two or four crystal detectors in the same encapsulate, connected using an arrangement anti-serial (serial connection, but with phase opposition). Applying this placement, similar internal temperature changes should be canceled due to phase opposition. Therefore, dual-element detectors have the inherent advantage that the output voltage is the difference between the voltages obtained from each pyro-element, which internally removes the thermal environmental changes effects.

**Figure 4:**
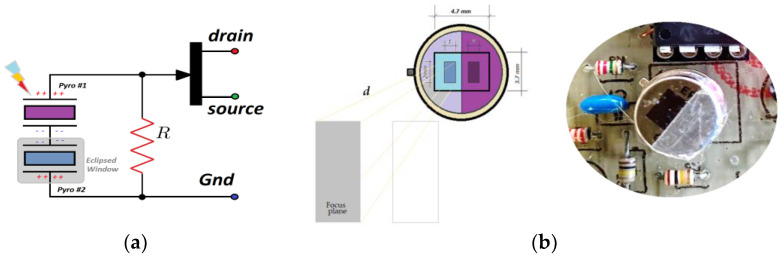
A dual-element PIR sensor: (**a**) the pyro-crystal and amplifier schematic. (**b**) PIR sensor displays the sealed one-half window of the sensor aperture by using an adhesive metallic sheet.

To obtain a maximum response with this arrangement, it is required to include a Fresnel lens that focuses the environment by facets and prevents the identical spatial areas focuses on both pyro-elements (Figure 4). This is the reason why the vast majority of commercial sensors use this lens method because they are primarily oriented to intruder detection.

**Figure 5:**
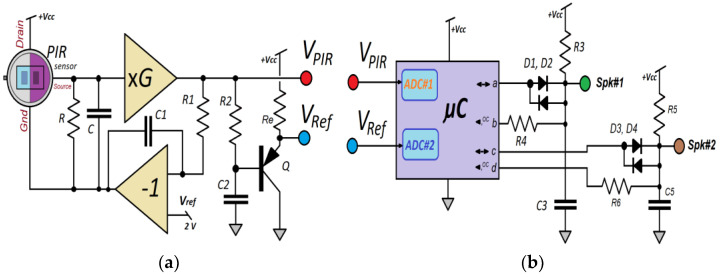
(**a**) Proposed circuit for the amplification of the output voltage provided by the PIR sensor: a time-varying voltage, *V_PIR_*, is obtained jointly with a central reference value output, *V_Ref_*. (**b**) To achieve pulse trains (emulating the spiking neuron), a fast algorithm destined to run on a small microcontroller is implemented.

This work proposes the use of a single sensor (eclipsing the second encapsulated element) (Figure 4b) positioning an adhesive metal foil at the exact point of the half-window’s division. In this situation, one of the pyroelectric crystals allows the detection of external heat, and the other pyro element compensates for the thermal drift of the sensor itself. To amplify the signal from the PIR output, an operational amplifier is used in a non-inverting configuration which provides a gain of around 40 dB (Figure 5a). The amplifier is also feed-backed utilizing an integrator that, after averaging the output voltage of the sensor for a 1.5 s period, modifies the polarization of the FET transistor integrated into the PIR sensor capsule (components *R*_1_ *and C*_1_, in Figure 5a, with a time constant of 1.5 MΩ ∙ 1 μF = 1.5 s).

It is interesting to generate a spiking output that allows the sensor to be connected to other spiking processing architectures to obtain a spike train with a variable period depending on the amplitude of the analog voltage provided by the PIR sensor. With this objective, different hardware topologies have been studied and assayed to generate this spike train. Given the size and cost of some current microcontrollers, a mixed analog and digital implementation was decided (Figure 5b), incorporating a low-cost microcontroller (the ATTiny85 device, from Atmel© (San Jose, CA, USA), now Microchip^®^ (Chandler, AZ, USA) [45]).

If the option of the microcontroller and its internal ADC is used, the procedure to pursue is the following:

1. The voltage generated by the PIR sensor is amplified through one operational amplifier (non-inverting). This output is again filtered using a passive RC network (*R*_2_ and *C*_2_, Figure 5a);
2. The filtered output after *R*_2_ and *C*_2_ now increments the offset level (due to emitter follower topology around transistor *Q*) about 0.6 V. This voltage, *V_Ref_*, higher than the quiescent *V_PIR_*, is the reference actual value of the *V_PIR_ − V_Ref_* conversion;
3. It is necessary to synthesize the pulse sequence from the difference (*V_PIR_* − *V_Ref_*). For this purpose, a difference equation characteristic of an IIR-type digital filter is established.

The algorithm of this filter must be derived from basic design principles, starting from the operation of the analog component required to implement a LIF (leaky integrated and fire neuron) spiking circuit, which, unlike other models, can replicate the spiking behavior of a biological neuron using a minimum number of circuit elements.

Since the neuron model proposed by Hodgking-Huxley [46], the literature has seen a significant increase in the number of implementation proposals based on discrete hardware techniques (transistors, logic gates, amplifiers, etc.) or configured circuits on FPGA (field programmable gate array) or CPLD (complex programmable logic devices). For example, it is interesting to know the background given in the paper by Dutta et al. [47] on a new method for implementing MOSFET-based LIF neurons.

**Figure 6:**
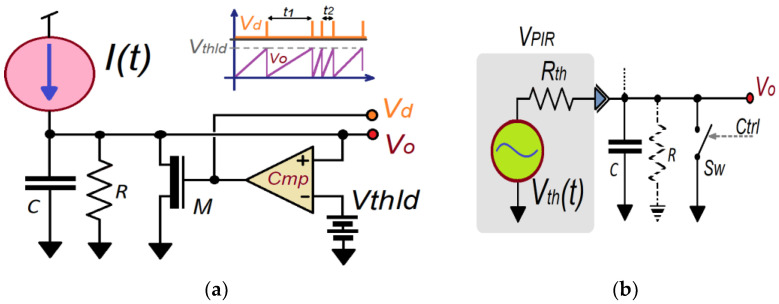
(**a**) Classical LIF (leakage integrate and fire) neuron hardware model and its equivalent, and (**b**), using its *Thevenin* equivalent to emulate using difference equations.

The starting point for the design of the LIF is a relaxation oscillator circuit based on the charging of a capacitor and the forced discharging through a non-linear element (in this case, across a transistor controlled by a comparison threshold) (Figure 6a).

The input to the LIF circuit is a current source. It represents the detected analog variable as a function of time. The model represents the neuron as a parallel combination of a resistor (conductance modeled as 1/*R*) and a capacitor *C* as shown in Figure 6.

Depending on the current, the capacitor will take more or less time to complete its charge. A comparator (with a fixed and determined threshold) observes when the capacitor charge has reached the comparison level (*V*_o_(*t*) > *V_th_*). If so, it forces the discharge (reset) of the capacitor *C*. Thus, the intervals (*t*_1_, *t*_2_, …) of the sequence of discrete pulses generated by the comparator depend on the input variable according to the below equation:

(2) $$C\frac{dVo}{dt}=\gamma \left(V_{C}\left(t\right)-E_{L}\right)+I\left(t\right)$$

where *E_L_* is the resting potential where the capacitor tends due to the transistor’s output impedance, *γ* the equivalent conductance, and the term *γ*(*V_C_*(*t*) − *E_L_*) is the modeled internal leakage current. *I*(*t*) represents the different neuron excitations from other spiking cells.

To a low-level input signal as a consequence of a low input current (*I*(*t*)), *V_C_*(*t*) never exceeds threshold *V_th_*, which produces no spikes during a long time interval (*t_i_* → ∞).

The leakage impedance is, in general, extremely high. For this reason, it is possible to simplify the model to:

(3) $$C\frac{dVo}{dt}\approx I\left(t\right)$$

Applying the *Norton–Thevenin* equivalent theorem, Equation (3) can be modified to adopt voltage changes due to the PIR output voltage rather than current changes. Figure 6b shows a LIF voltage-controlled model where the current source (*I*(*t*)), is now characterized by the *Thevenin* equivalent voltage source (*V_th_*(*t*) = *I*(*t*)∙*R_th_*, where this *Thevenin* impedance includes all circuit resistances). This voltage is the PIR signal output. In this case, the expression to represent the equation in differences is:

(4) $$\frac{dV_{o}}{dt}\equiv \frac{\left(V_{o}\left(n\right)-V_{o}\left(n-1\right)\right)}{T_{s}}=\frac{1}{C}\left(\frac{V_{PIR}\left(n\right)}{R_{th}}\right)$$

This difference equation may be simplified and expressed as:

(5) $$V_{O}\left(n\right)=\frac{T_{s}}{CR_{th}}V_{PIR}\left(n\right)+V_{O}\left(n-1\right)$$

where *n* denotes the time order of the present sample to *V_o_* and *V_PIR_*, and (*n −* 1) indicates the preceding sample of *V_o_. T_S_* is the sampling period imposed in the processing algorithm to run on the AT-Tiny85 microcontroller. The sampling rate should be set taking into account the bandwidth needed to process the PIR response as well as the speed of the elements to be detected within the observation scene. An appropriate choice of the factor (*T_S_*/*C*∙*R_th_*) as an integer will simplify the equation and make the algorithm more quickly computable. This is essential for real-time processing.

According to the Centers for Disease Control and Prevention data, the average walking pace is around 5 km/h. This information allows us to design the analog amplifier circuits and establish the constants within the processor. On the one hand, since the speed of 5 km/h corresponds to a value of about 2 m/s, the time intervals can be determined based on the activity of the subjects in front of the IR sensor. In [40], the authors consider velocity ranges between 0.27 m/s to 1.388 m/s. Therefore, 2.0 m/s may be a reasonable estimation.

To be able to discriminate changes in the position of the scene with a length of about 50 cm (at a speed of 2 m/s), times of approximately 0.25 s are required. This data establishes a frequency of about 4 Hz. This value fixes the passband upper corner frequency for the amplification stages. Further, a value of 4 Hz allows us to choose the minimum sampling frequency (about 10 × 4 Hz) for the voltage generated by the PIR sensors.

By approximating the constant *T_S_/C∙R_th_* to 4, it is possible to reduce the execution time of the algorithm since the multiply operation is configured as a double left shift. This value can be achieved by applying a sampling frequency of 40 Hz and choosing a theoretical RC network of 1 kΩ × 6.2 μF. With this, the LIF algorithm may be implemented from the following Equation (6):

(6) $$\left\{\begin{array}{c} \begin{array}{c} V_{O}\left(n\right)=4\left(V_{PIR}-V_{Ref}\right)+V_{O}\left(n-1\right)\\ V_{O}\left(n-1\right)=V_{O}\left(n\right) \end{array}\\ if\;Vo\left(n\right)>V_{thld}\;\underset{\;}{\Rightarrow }\;V_{O}\left(n\right)=0 \end{array}\right.$$

where *V_thld_* is the binary value to set the threshold spiking (about 85% of the microcontroller power supply), and *V_O_*(*n*) = 0 is the reset condition. Internal processor timers are used to synthesize the periodicity of the pulses. Programming using interrupts allows for exact synchronization of the firing rate calculation. The ATTiny85 microcontroller integrates two configurable timers to trigger interruptions at the end of the programmed count. TMR0 is dedicated to controlling the capture sequence of the analog inputs (*V_PIR_* in ADC0 channel and *V_Ref_*, in ADC1 one) to 100 Hz sampling frequency (*Fs*). This sampling period is shared with each channel giving an effective sampling frequency of 50 Hz per channel (*V_PIR_* and *V_Ref_*).

**Figure 7:**
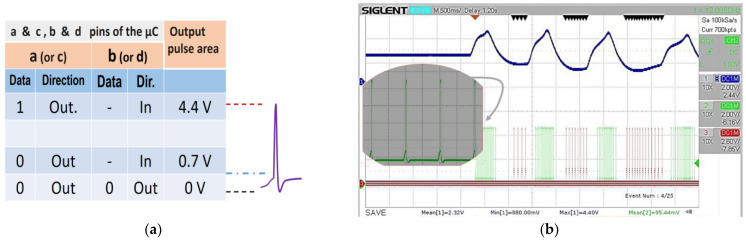
(**a**) Truth table to conform the shape of the analog spiking out (reconfiguring on fly the port direction of pins). (**b**) A view of the spike trains synthesized by the microcontroller, depending on the analog input value (PIR sensor voltage out).

The pulses are generated using only two digital outputs (Figure 5b). The out RC filter allows soft changes to the original discrete output. A dynamic reconfiguration of output pins and two diodes lets three-level analog states simulate the refractory spiking period: 0 V, 0.7 V, and 87% of Vcc (Figure 7a). The different events to synthesize the output spiking pulse shape are definable values (as variables) within the program execution in ATTiny85.

In this way, a pulse can be shaped for different conditions by essaying with other timing for each logic state described in Figure 7a. However, significant shaping changes may also require choosing different values for the capacitance and resistance of the *R*_3_, *C*_3_, and *R*_4_ (and *R*_5_, *C*_5_, *R*_6_) components (Figure 5b). An oscilloscope image of the spiking pulses generated by the processor, following the PIR voltage output dynamic changes, is shown in Figure 7b.

Since the voltage response of the PIR sensor is similar to a capacitor charge/discharge when a warm object moves away from the detector, a peak is also produced, but in this case, with a negative value. The same algorithm implemented by Equation (6) is also used to synthesize these additional spike trains. These new pulses (for the inverse operation) now denote the decreasing temperature perceived by the sensor. For this, it is necessary to invert the calculation of the PIR voltage concerning the reference one. The new implementation algorithm is below:

(7) $$\begin{array}{c} \left\{\begin{array}{c} \begin{array}{c} \begin{array}{c} If\;\left(V_{Ref}-V_{PIR}\right)>0\\ V_{1O}\left(n\right)=4\left(V_{Ref}-V_{PIR}\right)+V_{1O}\left(n-1\right) \end{array}\\ V_{1O}\left(n-1\right)=V_{1O}\left(n\right) \end{array}\\ if\;V_{1O}\left(n\right)>V_{thld}\;\underset{\;}{\Rightarrow }\;V_{1O}\left(n\right)=0 \end{array}\right. \end{array}$$

A new set of variables (*V*_1*O*_(*n*) and *V*_1*O*_(*n* − 1)) is declared to run the new algorithm in parallel, taking advantage of the interruption mechanisms by the microcontroller configurable timers.

It is well known that the synthesis of genuine spiking outputs in real-time applications requires a significant computational effort. It is necessary to model the biological behavior (generally described by differential equations) and to generate accurate waveforms (numerical, if the purpose is for inside processing or a proper analog output to produce external excitatory signals) [48].

To start the analysis, various emulations of firing pattern sequences based on the LIF-type spiking neurons were essayed. However, it is possible to take advantage of the proposed digital–analog interface (R, C, and diodes, in Figure 5b) to improve the shape of the pulses by choosing other component values.

This work is based on the Izhikevich study [49] to synthesize the analog spiking-out shape. It is considered a pulse’s maximum rate (firing rate) of about 100 Hz. Adjusting some figures from Izhikevich’s work, it was possible to adapt the spike patterns for a 5 V amplitude of the power supply. The firing pattern levels are characterized by a total amplitude of −70/−60 mV (resting potential) to a maximum of 30 mV (peak). This gives a range of about 100 mV. The threshold trigger potential is around −55/−40 mV.

Therefore, a range from 0 V to 5 V (TTL power supply) is conceivable to characterize approximately the biological amplitude: 30 mV − (−70 mV) = 100 mV. Thus, the voltage drop on a classical diode (about 0.7 V) would represent nicely, in this range, the firing threshold (about −50 mV in the biological neuron). To model the pulse shape, a list of constants indicating the interval of every pulse phase was introduced on the microcontroller code. Now, the event is characterized by 5 ms for the refractory period and 3 ms for pulse peak configuration. These values permit generating spike rates upper to 100 Hz (period 10 ms).

### 2.2. Summation Neuron Board

In the previous section, the design and implementation of infrared-sensitive cells have been depicted as a basic emulation of biological cells by applying a spiking procedure to their output pins. In this way, nerve impulses are simulated, where the detector’s captured parameter intensity is modulated from the temporal sequence of pulses. The output of the proposed circuits can then be seen as the cell *axon*; the wire bus that carries the signals from each PIR detector can be considered an optical nerve.

However, neural processing is needed now (as a soma equivalent emulation) that allows it to make sense of what is captured by the PIR sensors array. This board receives the postsynaptic potentials emulated from sensory neurons (PIR boards).

This is the spiking neuron’s specialty and the necessary training to make them useful. This field is fully developed, and one considerable number of treatises, references, and reviews on their fundamentals or about new frontiers of the research can be consulted. For example, it is possible to mention numerous references on the spiking neural networks area, including [36,50,51], only to cite just a few, or their implementation exploiting hardware techniques [52,53,54,55] or photonic procedure based [56] methods. The digital achievement of neurons in programmable devices is more than feasible, especially on FPGAs or fast processors. This has enabled today’s dense neural networks and deep learning. However, this work follows the implementation of basic networks, and therefore the techniques and resources must be adapted to more basic hardware. Therefore, since the core of the processing board will be a microcontroller, the best option is the implementation of neurons utilizing the usual IIR filter routines, based on the multiply and accumulating operation (MAC). This approach has been proposed in the Maass-Bishop text [57], as well as several articles also published at this time [58].

**Figure 8:**
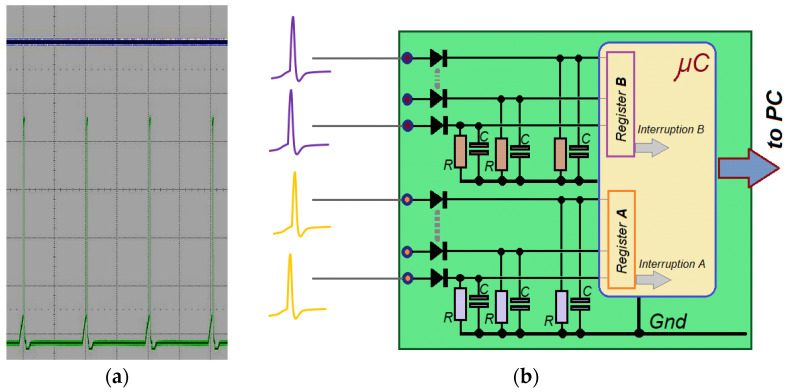
(**a**) An oscilloscope image of a spike train generated by the PIR sensor board. (**b**) Block diagram of the spike receiver circuit is based on an 8 bits microcontroller board, using up to 8 digital interrupt inputs in this prototype (four PIR sensors, two signals for detector).

To simplify the device, it was necessary to design a circuit that could operate digitally (avoid analog-to-digital conversions) but could successfully count the received pulses from the spiking neurons. The fastest processing technique is the usage of the interrupt services generally integrated into any microcontroller as control resources (Figure 8b). The usefulness of hardware interrupts is to speed up the routines to capture and count the acquired spikes, though it requires discretizing the waveform of the received pulses. It is necessary to detail that although the spike train advances with a frequency rate of about 100 Hz, the very narrow shape of the peaks will require considerable bandwidth to be processed. Therefore, the analog sampling option must be carried out with a high rate regarding the sampling frequency. For this, the analog sampling and acquisition choice for the different pulses leads to time delays that decrease the validity of this method.

Moreover, the pulse-rate estimating technique by interrupts can be implemented in configurable devices such as FPGAs or CPLDs (not only in microcontrollers). Although the number of inputs to the central module (summation neuron) depends on the processor inputs number, this quantity was limited to eight in this prototype: four inputs for every PIR detector and four input pins for the complementary out pulses (which are triggered when the source-sensor distance increases). In total, two pulse-carrying wires for each sensor neuron (PIR) and eight inputs in all.

The spiking receiver board core is an Atmel^®^ (San Jose, CA, USA) ATMega328 microcontroller with a processing speed of 16 MHz. It can support elementary processing routines for people detection and simple classification functions such as optical flow-based techniques. However, for other tasks that require a higher complexity, any platforms currently available on the market can be used, such as boards based on Atmel’s (Microchip^®^) XMega^®^ devices, ARM microprocessors, or ‘single board computer’ (SBC) platforms.

Since the pulses from neurons come with an offset of about 0.65 V–0.7 V characterizing the threshold voltage, the summation neurons acquisition circuit requires a diode to shift down the offset voltage (Figure 8b, to match the digital ground level of the processor board and the sensor boards). Resistors are also required to keep the processor inputs at a low level. The parallel capacitor sustains the input pulse (slowed discharge) to facilitate counting from the microcontroller interrupt mechanisms.

### 2.3. PIR Sensors Array Structure: Design and Construction

**Figure 9:**
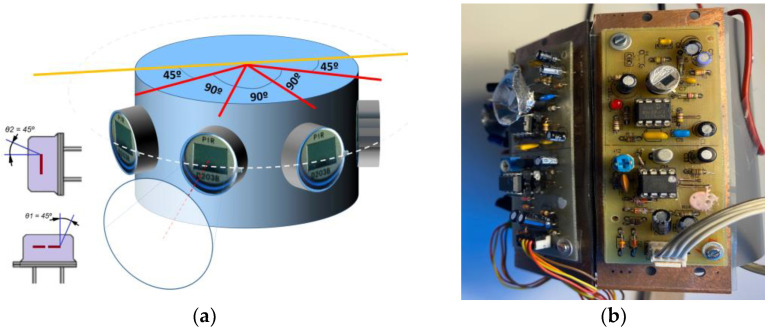
(**a**) A cylindrical arrangement for four PIR Sensors to extend a 180° field of vision. (**b**) Assembled prototype on a cylindrical surface.

Depending on the application, different structures can be formed using the designed PIR detectors. One possible solution was tested: a cylindrical base to measure optical flow in a spherical type area around the position of every PIR sensor (Figure 9a). Another could be a spherical disposition emulating the PIT organ.

According to the manufacturer’s specifications for the selected IRA-E700 PIR device, each sensor features an FoV of +45° to −45°, for each X and Y axis (Figure 9b). To cover a vision area of about 180°, four sensors must be aligned to 45° between each adjacent sensor. This will limit the FoV to 90° (45°–(−45°)) for the neighboring sensors. It is required to confine that field of view by adding a truncated cone over the viewing window of the PIR sensor. Said cone can be built from rolled metal sheet, maintaining the appropriate proportions to the diameter of the sensor (9.0 mm).

### 2.4. IR Radiometry for Optical Flow Detection

For the human body’s standard temperature (about 37 °C, but around 30 °C if we consider the temperature of the limbs), the radiation presents its maximum at the wavelength of 9.56 μm. It is easy to determine by applying Wien’s displacement law, where the wavelength peak, in micrometers, is located on *λ_pk_* = 2897/*T* (Kelvin). The Stefan–Boltzmann law defines the power radiated from a black-body according to its temperature. Considering the subject area, A, and the coefficient of emissivity, *ε*, it is possible to calculate the power radiated from Equation (8):

(8) $$P=A\varepsilon \sigma T^{4}$$

where *σ* = 5.67 × 10^−8^ Wm^−2^K^−4^ is the Stefan–Boltzmann constant. Applying the heat of the human body to Equation (8), it is then concluded a power of 477 W∙m^−2^, or 0.0477 W∙cm^−2^ (a 100% emissivity has been considered, that is, a genuine black-body model, and referred to the unit area). It can be assumed that *V_PIR_* is proportional to the thermal transitions but, as a magnitude, dependent on the inverse of the distance to the radiating body.

In this case, taking *Q* and *K* as constants to determine may be established:

(9) $$V_{PIR}=Q\Delta T\equiv K\frac{1}{d^{\alpha }}$$

where *d* is the distance from the source to the *PIR* sensor, and where *α* must be around 2 due to the inverse square law principle.

Mukhopadhyay et al. [40] concluded in an expression to relate the PIR voltage response with several parameters in an equation, which they experimentally fit. Using constants, the result can primarily be formulated as below:

(10) $$V_{out}^{i}=K\frac{t}{d^{1.5}}F\left(\theta_{i}\right)$$

where *V^i^_out_* is the sensor output as the subject traverses the *ith* sector (Fresnel lens) with central angle *θ_i_*, and *i* represents the sector of the sensor’s FoV that is currently active or being traversed. *F*(|*θ_i_*|) is a deduced expression to introduce the angular dependence of *θ_i_*, due to the Fresnel lens. The parameter *t* is inversely proportional to the speed of movement of the subject. Thus, the voltage output distance dependence is given by a factor of 1.5. Then, IR propagation with spherical coordinates along the beam axis of each sensor can be formulated. Without the Fresnel lens, it can be assumed that along the axis, the vector in the sense of $\hat{r}$ defines the radiation level, but this remains moderately constant for the spherical coordinates $\hat{\theta }\;\left(polar\right)$ and $\hat{\varphi }$ (azimuthal), depending uniquely on the sensor FoV. With this approach, the measurements made by the sensor array can be based solely on the distance to the plane and will depend relatively little on the beam angle. Numerical solutions for analysis and estimation of sensor data can be calculated using techniques different from soft computing (e.g., gradient method, absolute measurements, the radial distance from the sensor center, etc.).

### 2.5. Optical Flow Processing

Optical flow is the perception of the apparent motion of elements (bodies, surfaces, objects, etc.) located in a visual scene, yielded by the relative motion between an observer and the scenario and how it affects to sensed image. Today it is notably applied because it allows the detection and measurement of motion-related phenomena: from determining the rotation speed of a tornado from the received images to the detection of moving bodies into a scene or the relative motion of a subject.

**Figure 10:**
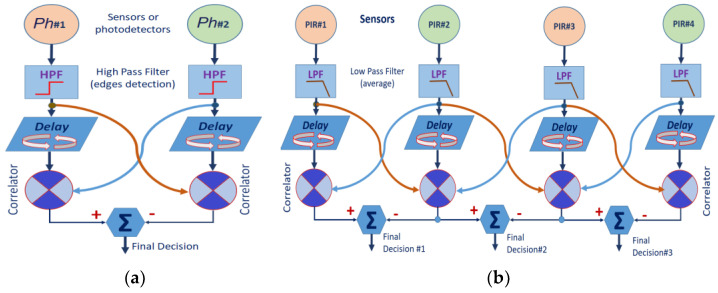
(**a**) Classical topology of the elementary movement detector (EMD) proposed by Hassenstein–Reichardt. (**b**) Architecture derived from the work of [55] with a linear network of EMDs.

Initially, optical flow theories were born in the 1950s, with works mainly by biologists, and initially focused on the analysis and knowledge of the insect’s compound eye and early vision. In this sense, the elementary correlational movement detector (EMD) formulated by Hassenstein and Reichardt deductions in 1956 is widely known and applied [59]. The procedure is based on an immediate principle of comparison, such as the behavior of the eyes of insects when they are exposed to fast luminous changes of any image elements from the scene [60,61] (Figure 10a). This model applies a correlation operator to the signal provided for one ommatidium with the delayed signal from a neighboring one. If the product of this correlation is maximum, this is due to a scene change event in the expected spatial sense, i.e., the direction from the second ommatidium to the first one. The final decision step takes the difference of every correlator outputs pair to determine the directional properties (regarding the sign and the module) and rejects the effects of temporal contrast not due to motion.

Some other works have been very relevant and even today continue contributing to scientific debate. In this case, the studies have opted for a matrix focus on the problem. (e.g., Lucas–Kanade, Srinivasan, Horn–Schunck, Buxton–Buxton, Black–Jepson, or general variational methods). Their techniques for optical flow detection have also been proposed specifically for use with computerized algorithms. The technical literature documents these techniques in a considerable number of books, articles, and references that point to all optical flow solutions achieved, as well as many triumphs by applying new machine learning techniques or involving new mathematical operators.

However, concerning the objective to implement solutions on small and low-power devices, the initial EMD technique can be a simple and affordable approach for the spiking neural network implementation around the previously analyzed PIR-type sensors. Current works confirm that the EMD correlated detector still has advantages in many respects and competes in particular instances with other types of optical flow computing solutions [62,63,64,65,66,67,68,69].

To take advantage of the results developed previously, a neural network approach to the problem is required, supported by the design of the PIR array that has already been presented. Given the hardware limitations of the processors used, it can be considered the ease of neural processing implementation with the benefit of DSP-type routines. Applying this method, it is possible to introduce the MAC (multiply and accumulate) algorithms characteristics of IIR and FIR filters. This way has already been proposed by [57] and in the work of [58].

Therefore, the generic optical flow proposal (Figure 10a) can be separated to carry it out by applying the resources available in the presented embedded platform. In this case, each ommatidium is a PIR sensor providing an output voltage, and its inverse voltage response, due to the dual behavior of the pyro-element capacitance, provides complementary information from every sensor.

Since the input to the integrated platform (that acts as the neuronal processing block) is a spike train, a low-pass filter must be implemented. This filter is necessary to integrate and shape the succession of peak excitations received from each PIR sensor.

On the other hand, it is considered that the sensors are spatially distributed around the cylinder surface. So, it is necessary to include the calculation of the correlation between adjacent detectors (lattice structure), as shown in Figure 10b.

Thus, with a set of N detectors, the analysis concludes in *N* − 1 individual optical flow results (*final decision#i*), two by two, whose resultant gives rise to an estimate of the total flow [55].

**Figure 11:**
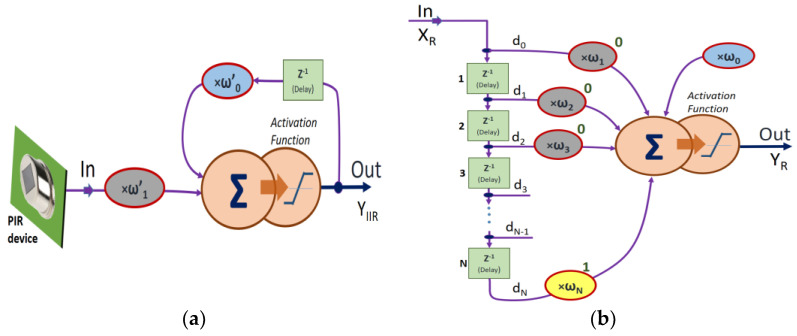
(**a**) Neural structure that responds to a low-pass IIR filter response, using a delay memory cell. (**b**) *Adaline* neural network configuration: in this case, it is used only as a significant delay network to emulate the behavior of the delay module of the Hassenstein–Reichardt (HR) EMD structure.

The first block to implement is the input low-pass filter, regarding it from a neural perspective (Figure 11a). The basic implementation algorithm of an IIR first-order low-pass filter can be obtained directly from the schematic of a simple RC integrator. In that case, the cutoff frequency given by:

(11) $$Fc=\frac{1}{2\pi RC}$$

The difference equation is then:

(12) $$y\left(n\right)=\alpha \cdot x\left(n\right)+\beta \cdot y\left(n-1\right)$$

where α = RCTs/1+RCTs and *β* = 1/1+RCTs.

For a maximum input spike rate of approximately 100 Hz (period *T* = 10 ms), a sampling frequency of about 10 Hz is required to facilitate the necessary calculations at that spike rate. We thus choose *T_s_* = 100 ms. To filter out motions represented after the sensor at voltage changes of about 1 Hz, a value of *RC* = 1/2*π* is required. Then, the values are *α* = 62/100 and *β* = 38/100.

To minimize the time computation these constants must be represented in integer and scaled to binary (base two). Thus, the divisions are converted into basic shifts. These numbers can be expressed as *α*~5/8 and *β*~3/8. If it considers that input filter from the perspective of a neuronal input, the input integration process can be performed as:

(13) $$Y_{IIR}=\left(\begin{array}{ccc} w_{1} & w_{2\;} & 0 \end{array}\;\begin{array}{ccc} \;0 & 0 & 0 \end{array}\right)\left(\begin{array}{c} Xin_{1}\\ Z^{-1}\left(Y_{1}\right)\\ \begin{array}{c} .\\ .\\ 0 \end{array} \end{array}\right)$$

Then, the delay block on every signal branch of each ommatidium must be entered into the computation according to the operation of the EMD. This function may be seen as an *Adaline* (adaptive linear element) network working as a delay line structure [70]. The basic implementation can be seen in Figure 11b. A ‘circular buffer’ allows the data rotation and obtains a considerable delay according to the sampling frequency set for the algorithm. It is possible to see the delay network implemented as another neuron of the network, this one, with the *Adaline* structure. To achieve a given delay, it is uniquely required to select that corresponding weight at a non-zero value, while the rest are defined at a null value. This results in the following expression, which determines the delayed output:

(14) $$Y_{R}=\left(\begin{array}{ccc} 0 & 0 & 0 \end{array}\;\begin{array}{ccc} \;0 & 0 & w_{N} \end{array}\right)\left(\begin{array}{c} d_{0}\\ d_{1}\\ \begin{array}{c} .\\ .\\ d_{N} \end{array} \end{array}\right)$$

where *d_i_* follows the sequence of delays as shown in Figure 11b.

**Figure 12:**
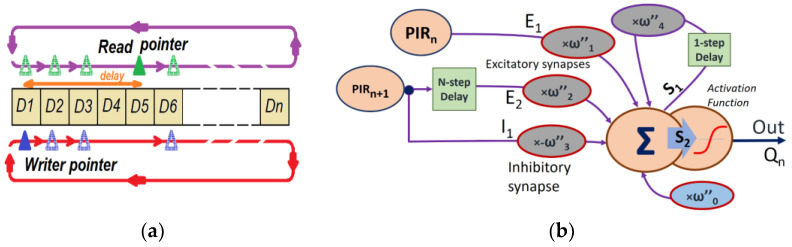
(**a**) Circular buffer software implementation, making use of dual pointers (writer a read data). (**b**) A complete realization of the HR EMD optical flow based on a neural network applied to two adjacent sensors.

The algorithm to solve the ‘circular buffer’ consists of moving two out-of-phase pointers to a certain number of positions over an array of data in the random access memory (RAM) (Figure 12a). The offset between the write pointer (*P_W_*) and the read pointer (*P_R_*) introduces a delay according to (*P_R_* − *P_W_)* × *Ts*, with *Ts* the sampling period. An accurate estimation of the necessary delay requires knowledge of the exact velocity range of the subject and the distance to the sensor. From the FoV angle of each sensor, it can be determined when the subject is no longer projected on sensor #*n* to activate the adjacent sensor #(*n* + 1). To begin the tests, a delay of 3 to 4 steps has been considered, which for a *Ts* = 100 ms covers delay intervals between 200 and 400 ms between sensors.

Finally, it is necessary to process the correlation between direct and delayed signals. The more basic correlator consists of a simple product of both signals. This operation is performed at the rate set by the sampling period *Ts* from the immediate data obtained from successive calculations. The integer multiplication operation does not require an excessively high computational speed, but it can be a bottleneck for operations to be executed on a low-speed microprocessor. Therefore, this work proposes a method to compute the correlation with the help of a neural network, taking advantage of setting different weights for every input, including negative values as weights.

Inputs with negative weights will act as inhibit inputs. In this case, the last neuron sums the signals coming from the *n* sensor output, and the adjacent, *n* + 1, is delayed to check the moving sense of the subject in the FoV.

The multiplication of the correlation operator greatly maximizes the situation that both signals are in phase. This is not the case for a simple sum of signals. To enhance the difference, the direct signal of the delayed sensor itself is subtracted from the corresponding signals. Thus, this negative weight helps to reduce the sum value of the neuron when there is effectively no correlation between adjacent sensor signals (actuating as IR pixels). This input actuates like an inhibitory synapse. In addition, a sigmoidal activation function has been imposed to improve the situation when an overlap occurs between the direct and delayed signals of two sensors.

For the real-time implementation of the sigmoidal function, a discrete figure (in integers numbers) $S\left(x\right)=1/\left(1+e^{-x}\right)$ constrained to the binary range 0 to 255 in both X and Y axes has been introduced as a LUT (*Lookup Table*). This function has been tabulated with the help of the free software GNU OCTAVE. These values, calculated using $S_{B}\left(n\right)={\left.round\left(\frac{255.0}{1+e^{-kn}}\right)\right|}_{-128}^{128}$, were entered into the microcontroller program memory as a 256-row table.

The basic EMD HR topology (Figure 10a) can be implemented together, as shown in Figure 12b. This topology corresponds to the matrix operations below:

(15) $$\left(\begin{array}{cccccc} {\omega^{\prime \prime }}_{1} & {\omega^{\prime \prime }}_{4} & 0 & 0 & 0 & 0\\ 0 & 0 & 1 & {\omega^{\prime \prime }}_{1} & {\omega^{\prime \prime }}_{3} & {\omega^{\prime \prime }}_{0} \end{array}\right)\;\left(\begin{array}{cc} PIR_{n} & 0\\ Z^{-1}\left(S_{1}\right) & 0\\ 0 & S_{1}\\ 0 & Z^{-M}\left(S_{1}\right)\\ 0 & PIR_{n+1}\\ 0 & 1 \end{array}\right)=\left(\begin{array}{cc} S_{1} & 0\\ 0 & S_{2} \end{array}\right)$$

where *Z*^−1^ (*Z* transform) acts as a delay operator and *Z^−M^* an *M*-step delay operator. *S*_2_ is the neural output sum before applying the sigmoidal activation function. *PIR_n_* represents the output voltage to a sensor in particular, and *PIR_n+_*_1_ is the adjacent sensor output. The weights *ω″_i_* have been calculated following the procedure of the IIR filters already explained. The weights adjustment, particularly of *ω*_0_, could be optimized by applying a training process to the neural network to a given application.

Equation (15) refers to a single element: it must remember that the final result requires the processing of *N* − 1 individual results *Q_i_*, for *N* sensors, analyzed two by two (Figure 10b). The sum of all correlated outputs for every direction is added together (in this case, *N* − 1 operations for each axis). The average of all directional resulting components is subtracted to find the overall displacement value of the entire array.

However, it is possible to efficiently introduce the processing in a matrix method, programming it into a FOR-like structure that sweeps from 0 to the maximum number of sensors, *N*. The corresponding code has been written in the C language and downloaded on the 16 MHz microcontroller ATMega328 board.

## 3. Case of Study

**Figure 13:**
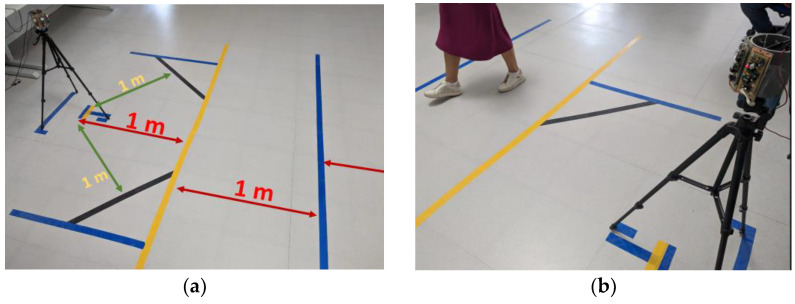
Tests on marked soil: (**a**) labeled plant and (**b**) standard camera tripod supporting the sensors assembly.

As a study case and using the constructed PIR array (Figure 9), an optical flow application will be implemented. The four sensor boards have been adjusted and cylindrically assembled following the previous description. They have been screwed to a rigid PVC cylinder to maintain a structure that allows an FoV range of 180°. The measurements were made in a large room to avoid multiple IR reflections of uninteresting objects in the observation scene. To compare the algorithmic results according to the subject path and sensors distances floor was marked at distances of 1, 2, and 3 m around the sensor assembly (Figure 13a,b).

Three tests and one calibration have been performed to determine the validity of the circuit complete (sensors and postsynaptic emulation).

First, an adjustment procedure must validate a quasi-linear response for every sensor module (presynaptic neuron). This test requires a direct excitation from the DC voltage source and a substitutive of the PIR output. A sweep from 0 to 5 V should test the spiking output ratio (up to 100 Hz approx.) according to the algorithm represented by Equation (6). The behavior for the application of Equation (7) has been checked in the same way.

**Figure 14:**
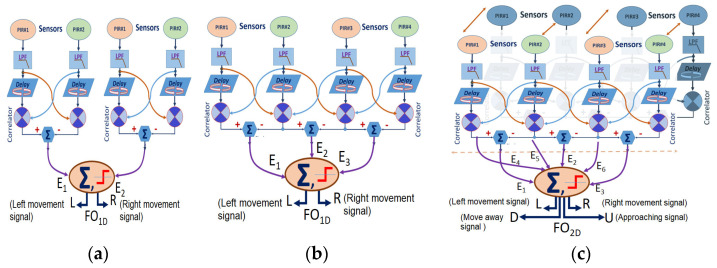
Three neural optical flow algorithms implementations: (**a**) four-elements, two-by-two; (**b**) four-element lattice array; and (**c**) eight-element array considering the complete information (positive and negative output) provided by each PIR sensor.

The first and second tests aim to check the optical flow algorithms based on the proposed neural computation. For the simplest case (two sensors), the problem is reduced to a two pixels optical flow calculation. It assumes the output of two independent EMDs (Figure 14a), calculating an optical flow result from both EMD branches. In this case, the result sufficiently differentiates the activity in front of sensors #1 and #2 (about 90° from the FoV) and the other set (sensors #3 and #4) for the rest of the scene.

The second test has considered a lattice algorithm that provides outputs in both paths. Since there are four sensors in the array, only three equations of motion can be determined (Figure 14b). The resultant is obtained from the sums of all calculated correlations (vectors) to the right and the sum of all vectors to the left.

To perform the correlation procedure, the tests executed employing the multiplication operation are more directly resolute since this product favors numerical discrimination when the visual signals (direct and delayed) are in phase. However, genuinely neural processing gives it a particular focus, helping inhibitory functions, as well as biological systems.

Finally, a different lattice algorithm was estimated that provides four computed outputs. A computational block is dedicated to the lattice optical flow from sensors #1 to #4. A different routine calculates the pairwise optical flow between every sensor #n and itself, coming from its other negative output voltage (Figure 14c). Looking now at the spike train receiver circuit Equation (10) must be considered, which defines the inverse dependence of the delivered PIR voltage as a function of the distance from the subject.

## 4. Results

The results computed inside the ATMega328 microcontroller (postsynaptic neuron) have been externally monitored using a USB connection to a laptop and oscilloscope-like software on the computer. The adjustment and calibration procedures were carried out on the bench utilizing an oscilloscope and a function generator.

To determine greater distances with this type of sensor, the gain value of the amplifier stage is crucial. For the prototype presented, it has been decided to simplify and implement a single amplifier stage. However, this circuit topology allows more amplifier stages to be easily included without excessively increasing power consumption or physical dimensions.

The first test (Figure 14a architecture) shows that the sensor behaves like a movement detector that covers 180° but is discretized in the 0°–90° and 90°–180° zones.

**Figure 15:**
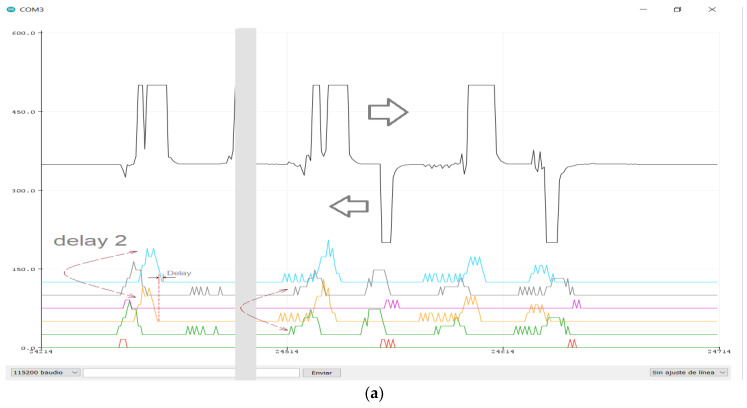

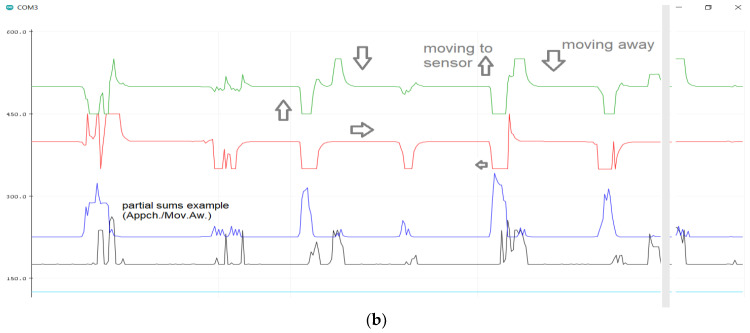
Optical flow detection: internal signals visualized using the Arduino IDE, Serial Plotter tool; (**a**) 1D, moving left to right (or vice versa) in front of the sensors (resultant vector of L and R signals, Figure 14b). (**b**) Combined displacement in different directions (resultant vectors of L, R, U and D signals, Figure 14c).

Test number two allows checking a continuous detection when the person walks circularly in front of the sensor array. However, the behavior is similar to the first test for walks perpendicular to an adjacent pair of PIR sensors. The output of the algorithm is displayed in Figure 15a. Positive peaks above the signal mean value indicate any warm object presence, moving in the rightward direction of the sensor array plane. The figure also shows the equivalent signals of each PIR sensor and their respective replicas delayed by the “circular buffer” function.

For the third test, the algorithm must take into account the spiking auxiliary output concerning the approach/moves away from every PIR sensor. The result is a kind of double-density array due to the double output of every sensor. However, discriminating increases/decreases of the pulse trains by approaching/moving away concerning lateral movement in front of the sensor requires a more sophisticated development of the algorithms. Figure 15b shows the evolution of a target in front of the sensor array. Two movement directions (left/right and approach/moving away) have been included in the processing. This last figure shows two flows computed for the two main directions. An external non-neural algorithm is necessary to conclude the definitive movement vector.

Table 1 shows some runtime measurements. These measurements were made with a 1 GSa/s, 100 MHz, 4 channels digital storage oscilloscope on the microcontroller platform, and any available output pins as pass-through flags indicators for the routines. The programming was carried out in C language. The clock frequency of the processor was 16 MHz.

## 5. Discussion

Several techniques have been confirmed to successfully execute optical flow calculations in real-time applications. Many of these works have been implemented on computers, graphics cards (GPUs), and FPGA development boards. These methods have also been tested with DVS (dynamic vision sensor) cameras for high-speed event processing. Therefore, the processing resolution comparison of this proposal concerning more complex systems is useless.

In favor of this method are the advantages of low-cost, low-power consumption, and reduced dimensions. In this sense, it should be noted that this low power requirement and an electric supply only from batteries will allow mobile applications or acting in remote locations (mobile robotics, sensor networks, ubiquitous computing, etc.). These features make it proper for low-level applications, mobile micro-robots, or automation applications, such as automatic doors that anticipate the movement of subjects.

Certainly, by applying the Fresnel lens, it is possible to achieve higher gain because the amplified sensor presents a discrete output. For a PIR linear implementation, it is not feasible to increase the amplifier gain in excess due to dynamic range: it would be required to develop a kind of electronic iris actuating on IR sensor gain.

## 6. Conclusions

An alternative proposal has been presented for the exploitation of the voltage produced by a PIR sensor concerning the detection of a subject within a scene, but taking advantage of its capacity as an analog sensor (avoiding the discretization introduced by the use of the Fresnel lens). In addition, a solution is provided and explained to obtain a pulsating output (to emulate the spike trains) for this sensor that presents a linear behavior depending on the infrared radiation it captures. This circuit itself represents an attractive proposal to convert any other type of linear output sensor on spike events. As a first conclusion, we can state that the procedure is valid for this type of sensor, as well as for other devices that require spiking processing of their output.

An unusual technique for matrix processing of the neural network based on digital signal processing techniques and IIR filters has also been introduced.

To verify the usefulness of transmitting the spike trains from the sensor to the electronic processing circuits, a primary technique for optical flow detection has been implemented (a study case). This has been developed using language C routines on an ATMega© 328 microcontroller core. Basic interface hardware with the sensors is added to adapt the coming signals to the electric characteristics of the microcontroller inputs. Even so, the design remains small in hardware size for the intended application which is optical flow detection. The tests demonstrate that the method is an efficient basic processing solution destined for low-level applications, using a spike train viewpoint without resorting to more complex processing platforms.

Regarding simple applications with PIR sensor arrays, where processing speed is not essential, the main limitation is sensing distance. The typical use of PIR sensors (Fresnel lens based) allows the implementation of high-gain amplifiers where saturation is not a difficulty but a benefit. In this application, a linear behavior of the circuits is required, and a limited value of gain restricts the detection distance.

On the other hand, the assembly of PIR higher-density arrays will require a processing speed increase due to the expansion in the number of outputs from the sensors (simulated optic nerve). Additionally, this output enlargement will require some multiplexing method to limit the number of wires until the postsynaptic processing circuit.

## Figures and Tables

**Table 1:** Execution times for different calculation routines presented.

16 MHz Processor Routine	4 Sensors	8 Sensors
Spiking detection (Interruptions)	9 μs	9–12 μs
Circular buffer	18.2 μs	18.6 μs
EMD (double pairing)	57.2 μs	--
EMD (lattice)	71.6 μs	100.4 μs

## Data Availability

Data (design information) available on request due to restrictions.
